# University of Louisville

**Published:** 1849-05

**Authors:** 


### UNIVERSITY OF LOUISVILLE.

Several very important changes have occurred in the medical department of the University of Louisville since our last number was issued, calculated, we think, to enhance the usefulness of the institution. We are sure that our medical friends, throughout the south and west, will be pleased to learn that the Trustees have secured the valuable services of Prof. E. Bartlett for the important chair of Theory and Practice. This gentleman has won an enviable distinction in Europe as well as America, by his valuable contributions to medical literature. His work on Fever has few rivals in this country, and has conferred no ordinary celebrity on its author. All his writings are pervaded by a spirit of philosophy, and are remarkable for the classical elegance and purity of their style. His mind is eminently practical, and, as an oral teacher of medicine, he is not less deservedly popular than as a writer. Prof. Bartlett has signified his acceptance of the appointment.

Prof. Yandell has been transferred to the chair of the Institutes of Medicine, lately held by Dr. Caldwell, and is succeeded by Benjamin Silliman, jr., Professor of Applied Chemistry in Yale College. Prof. Silliman's name has been most creditably associated with chemical science for a number of years; and, in addition to his wide-spread fame, the Board of Trustees had before them a number of letters from distinguished chemists in Europe and America, bearing strong testimony to his eminent abilities. He has been a successful teacher of chemistry in New Haven, and has received the warmest testimonials of the approbation of his classes. He has been for several years before the public as the assistant editor of the American Journal of Science and Arts, so well known as one of the most ably conducted scientific journals of the day. And, in addition to these evidences of his merits, he has published one of the best elementary works on chemisty which we have ever read.

May we not venture to congratulate the friends of the Louisville medical school, and of medical science, upon these auspicious changes? That they will add to the reputation of the school, and exert a beneficial influence upon medical science, we entertain no kind of doubt. With a Faculty thus constituted, and the new hospital arrangements made by the city Council, to which we have referred in another place, we cannot doubt that the institution is destined to a long course of prosperity and usefulness.

